# Eosinophils and COVID‐19: Insights into immune complexity and vaccine safety

**DOI:** 10.1002/clt2.70050

**Published:** 2025-03-22

**Authors:** Wided Sahli, Joana Vitte, Benoit Desnues

**Affiliations:** ^1^ Aix Marseille University MEPHI Marseille France; ^2^ IHU‐Méditerranée Infection Marseille France; ^3^ Laboratory of Immunology University Hospital of Reims Reims France; ^4^ INSERM UMR‐S 1250 P3CELL University of Reims Reims France

**Keywords:** COVID‐19, eosinophils, inflammation, ribonucleases, tissue infiltration, vaccination

## Abstract

**Background:**

COVID‐19 exhibits a variety of symptoms and may lead to multi‐organ failure and death. This clinical complexity is exacerbated by significant immune dysregulation affecting nearly all cells of the innate and adaptive immune system. Granulocytes, including eosinophils, are affected by SARS‐CoV‐2.

**Objectives:**

Eosinophil responses remain poorly understood despite early recognition of eosinopenia as a hallmark feature of COVID‐19 severity.

**Results:**

The heterogeneous nature of eosinophil responses categorizes them as dual‐function cells with contradictory effects. Eosinophil activation can suppress virus‐induced inflammation by releasing type 2 cytokines like IL‐13 and granular proteins with antiviral action such as eosinophil‐derived neurotoxins and eosinophil cationic protein, and also by acting as antigen‐presenting cells. In contrast, eosinophil accumulation in the lungs can induce tissue damage triggered by cytokines or hormones like IFN‐γ and leptin. Additionally, they can affect adaptive immune functions by interacting with T cells through direct formation of membrane complexes or soluble mediator action. Individuals with allergic disorders who have elevated levels of eosinophils in tissues and blood, such as asthma, do not appear to be at an increased risk of developing severe COVID‐19 following SARS‐CoV‐2 infection. However, the SARS‐CoV‐2 vaccine appears to be associated with complications and eosinophilic infiltrate‐induced immunopathogenicity, which can be mitigated by corticosteroid, anti‐histamines and anti‐IL‐5 therapy and avoided by modifying adjuvants or excipients.

**Conclusion:**

This review highlights the importance of eosinophils in COVID‐19 and contributes to a better understanding of their role during natural infection and vaccination.

## 1 INTRODUCTION

To effectively address a potential pandemic, it is vital to comprehensively grasp the various immune system mechanisms engaged upon virus infection. This knowledge fosters a better understanding of the pathophysiology and may initiate therapeutic avenues aiming at controlling viral replication, promoting virus clearance and avoiding/controlling infection‐induced immunopathologies.^1^ Despite extensive work, information regarding the immune response caused by SARS‐CoV‐2 remains incomplete.^2^ The release of pro‐inflammatory cytokines by innate immune cells is crucial in combating SARS‐CoV‐2 infection. However, this activation appears dysregulated during COVID‐19.^1^, ^3^, ^4^ In most infected patients, eosinophil counts are notably reduced, regardless of disease severity; however, this decrease is significantly more pronounced in severe cases.^5^, ^6^, ^7^, ^8^, ^9^ Moreover, recovery of eosinophil levels in COVID‐19 patients are linked to fewer critical care admissions and deaths.^10^, ^11^ Alterations in their surface molecule expression during infection suggest an early involvement in the immune response.^5^, ^8^ Although the specific roles of eosinophils remain largely unknown, their impact on the severity of natural infection and vaccine response is an ongoing matter of debate.^12^, ^13^

## 2 PATHOPHYSIOLOGY AND CLINICAL FEATURES OF SARS‐CoV‐2 INFECTION

**FIGURE 1:**
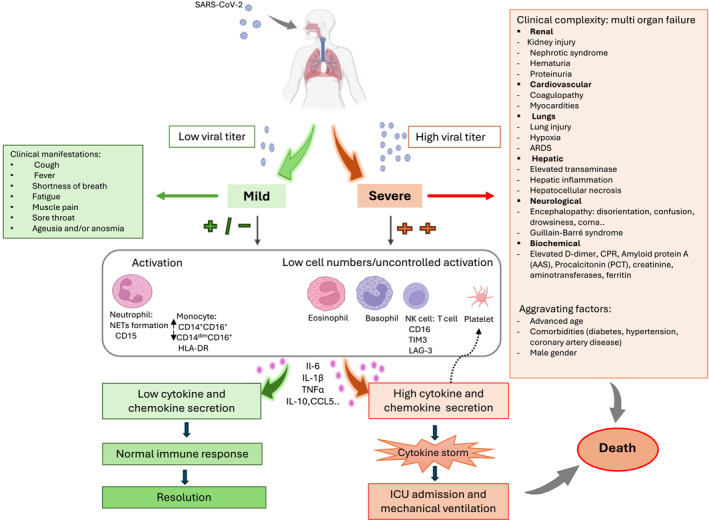
Clinical and immunological characteristics associated with COVID‐19 disease. COVID‐19 causes clinical manifestations that reflect disease severity, including respiratory and systemic complications. These manifestations depend on viral load and predisposing factors such as comorbidities and advanced age. Disease severity is often associated with acute immune activation, resulting in a cytokine storm. Conversely, a decrease in eosinophils, basophils, and lymphocyte counts remains a key characteristic of the disease. The exacerbated inflammatory state predisposes patients to an increased risk of admission to intensive care units, whereas a normal immune response leads to a rapid disease resolution without aggravation (see text for details).

SARS‐CoV‐2 infection is linked to manifestations of symptoms akin to those seen in the main respiratory viruses.^14^ Among the most commonly reported signs of COVID‐19 are cough, fever, shortness of breath, fatigue, respiratory distress, muscle pain, sore throat, ageusia, and/or anosmia.^15^ The trajectory of COVID‐19 can evolve from an initial asymptomatic or mild presentation to a critical state necessitating intensive care unit admission, oxygen therapy, and mechanical ventilation^16^ (Figure 1). Deteriorating outcomes are usually linked to the advancement toward acute respiratory distress syndrome (ARDS), resulting in pulmonary disability and severe hypoxemia.^17^ However, regardless of respiratory impairments, severe manifestations of the disease can encompass organ failure, myocarditis, coagulopathy, renal insufficiency, gastrointestinal disorders and neurological complications.^18^ In addition, autoimmune neurological syndromes such as Guillain‐Barré syndrome have also been reported.^19^ Hepatic dysregulation manifests as elevated transaminase levels, hepatic inflammation, hepatocellular necrosis, and exacerbation of pre‐existing liver conditions.^20^, ^21^ However, these complications remain limited in certain individuals with predisposing factors.^22^ COVID‐19 can also lead to various renal impairments, including drug‐induced acute kidney injury, thromboembolic, hypoxemic, and inflammatory causes.^23^ Other renal issues may encompass nephrotic syndrome, hematuria, and proteinuria.^24^ Deadly outcomes may manifest in individuals who are particularly vulnerable due to age or underlying medical conditions.^25^, ^26^ Various studies have consistently identified factors such as advanced age, male gender, hypertension, coronary artery disease, and diabetes as significant risk factors for severe COVID‐19 cases.^27^, ^28^, ^29^, ^30^ Biochemical abnormalities observed in COVID‐19 patients include markedly elevated levels of D‐dimer and amyloid protein A (AAS) among non‐survivors, as well as increasing levels of C‐reactive protein, procalcitonin, creatinine, aminotransferases and ferritin associated with worsening disease severity.^6^, ^31^ SARS‐CoV‐2 can cause severe disease in pregnant women, especially late in pregnancy,^32^ leading to complications like premature delivery, preeclampsia, increased cesarean sections^33^ as well as stillbirth and neonatal pathologies.^34^, ^35^

In conclusion and due to the complexity of its clinical manifestations, COVID‐19 can be considered a multi‐faceted condition. Indeed, SARS‐CoV‐2 is responsible for cellular disturbances affecting multiple organs, which can vary depending on host‐related parameters, such as pre‐existing conditions, or on virus‐related disruption of homeostasis.

## 3 IMMUNE DYSREGULATIONS ASSOCIATED WITH COVID‐19 SEVERITY

### 3.1 Major immune cell involvement

Several studies have noted a reduction in lymphocyte count in severe COVID‐19 cases^6^, ^26^, ^36^ and a close association between the decline in circulating lymphocyte counts and disease severity has frequently been described,^6^, ^37^, ^38^, ^39^ becoming a notable and persistent characteristic of COVID‐19 infection even after recovery.^40^ Previous research indicated that SARS‐CoV has the ability to infect lymphocytes.^41^ A similar process has also been observed during SARS‐CoV‐2 infection; however, lymphocyte infection appears independent of the ACE2‐Spike complex^42^ and rather involves the CD4 receptor,^43^ potentially contributing to lymphopenia observed during SARS‐CoV‐2 infection.

SARS‐CoV‐2 infection also disrupts the innate immune response.^26^, ^44^ In vitro, SARS‐CoV‐2 infects macrophages without replicating and triggers the release of both pro‐ and anti‐inflammatory cytokines, including IL‐6, TNF, IL‐10, IL‐1β, and TGF‐β, while IFN‐β production is notably absent.^44^ These cytokines, typically among the first to be secreted during viral infections, are markedly impaired during COVID‐19,^45^ suggesting an exaggerated inflammatory response following the initial virological phase.^45^ Additionally, thrombocytopenia has been linked to disease severity and mortality risk in SARS‐CoV‐2‐infected patients,^6^, ^46^ as previously shown in SARS‐CoV^47^ and MERS‐CoV^48^ infections. Immune thrombocytopenia affects all age groups, including children, adults, and the elderly.^49^, ^50^, ^51^ The decreased platelet count is associated with disease mortality^52^ and is attributed to lung tissue damage and pulmonary endothelial cell injury, resulting in platelet activation, aggregation, increased consumption, and reduced production within the lungs.^47^ The cytokine storm and autoantibodies may also contribute to the decline in platelet levels, although a production defect due to the virus's ability to attack progenitor cells cannot be ruled out.^53^ Monocyte counts exceeded normal levels in COVID‐19 patients.^54^ However, the frequency of HLA‐DR^+^ monocytes and their subsets differed among the study groups.^55^, ^56^, ^57^ Intensive care unit‐admitted subjects were characterized by a high prevalence of intermediate monocytes (CD14^+^CD16^+^) producing IL‐6.^58^ High and sustained levels of IL‐6 have been linked to mortality in SARS‐CoV‐2 infection, suggesting its potential as a therapeutic target for severe cases of COVID‐19.^6^ A reduction in non‐classical (CD14^dim^CD16^+^) monocytes also marked the severe forms,^57^ which was associated with a reduced transition from the classical (CD14^+^/CD16^‐^) to the non‐classical subset in critical COVID‐19 patients.^59^ Among individuals with post‐COVID‐19 sequelae, a consistent presence of CD169^+^ monocytes lacking HLA‐DR expression has been observed, emphasizing their potential contribution to sustained inflammation following infection.^56^ Interestingly, SARS‐CoV‐2 is able to infect monocytes, leading to an M2‐type polarization and an anti‐inflammatory response profile, which is also associated with antigen presentation deficiency due to low expression of HLA‐DR.^44^

Moreover, SARS‐CoV‐2 infection is characterized by reduced expression of CD16 (FcγRIII) in neutrophils, NK cells, and immature granulocytes.^55^ In severe COVID‐19 cases, there is a notable decrease in NK cells expressing CD16 compared with healthy individuals.^55^ This decline in CD16 expression may not be related to NK cell depletion but rather to a downregulation of CD16 as NK cells also possess other activator and inhibitor receptors like NKG2A, NKG2C, NKp30, and NKp46.^55^, ^60^ Besides receptor internalization, additional mechanisms also contribute to reduced CD16 expression on NK cells, including transcriptional downregulation.^55^, ^61^ After IL‐12 stimulation, CD56^dim^ NK cells also displayed reduced expression of CD16.^62^ Of note, IL‐12 is highly secreted during SARS‐CoV‐2 infection.^3^ Additionally, during influenza vaccination, IgG‐mediated immune complexes induce CD16 downregulation, potentially moderating immune responses.^63^ Impaired function of NK cells, indicated by increased TIM3 and LAG‐3 expression, may contribute to their exhaustion in severe COVID‐19 cases.^64^

Severe COVID‐19 patients also exhibited elevated neutrophil levels compared with healthy donors, alongside reduced CD15 expression in the most severe cases.^55^ Previous studies have linked extensive neutrophil infiltration into the lungs and increased peripheral blood neutrophil counts with the development of acute lung injury, ARDS, and mortality in SARS and MERS infections.^65^, ^66^ During COVID‐19, neutrophils form neutrophil extracellular traps (neutrophil extracellular traps) that promote thrombosis, leading to worsening of tumor states and chronic diseases, and contributing to pulmonary and neurological disorders.^67^ Neutrophils and macrophages are the primary immune cells driving the cytokine storm in the lungs of severe COVID‐19 patients.^3^, ^64^ Elevated IL‐6 levels, seen in SARS and MERS infections,^68^ are also prominent in COVID‐19.^6^, ^64^ Myeloid cells, including monocytes and dendritic cells, exhibit reduced expression of HLA‐DR and CD86, critical for antigen presentation to T‐helper cells, in severe patients,^69^ attributed to IL‐6 overexpression, contributing to immune dysregulation.^70^

Regardless of disease severity, eosinopenia was evident in the majority of patients upon admission.^27^ In both severe and non‐severe cases, there was a positive correlation between eosinophils and lymphocyte counts.^27^ This implies a potential interplay between these two cell types, fostering suppressive effects, which may elucidate their early depletion as characteristic markers of COVID‐19.^4^ Normalized lymphocyte, eosinophil, and platelet levels may signal recovery, whereas rising neutrophil, basophil, and IL‐6 levels correlate with increased risk of death in COVID‐19.^6^, ^10^, ^11^

Cytokines such as IL‐6 and IFN‐γ serve as predictors of disease severity, showing a downward trend from initial hospital admission to recovery. Additionally, they exhibit a negative correlation with humoral responses.^71^ The presence of neutralizing autoantibodies against type 1 IFN is linked to decreased levels of IFNα, particularly affecting men, and is associated with a worse prognosis.^72^ Patients have been found to possess these neutralizing autoantibodies prior to infection, establishing a predisposition for severe forms of COVID‐19.^72^ Mutations in genes responsible for IFN synthesis, such as TLR3 and IRF7, could also account for the reduction in type 1 IFN levels.^73^ Hence, deregulation of the immune response in COVID‐19 affects both innate and adaptive immunity, leading to functional modifications which affect the severity of the illness and seem associated with host‐related factors.

### 3.2 Eosinophils and SARS‐CoV2 infection

**FIGURE 2:**
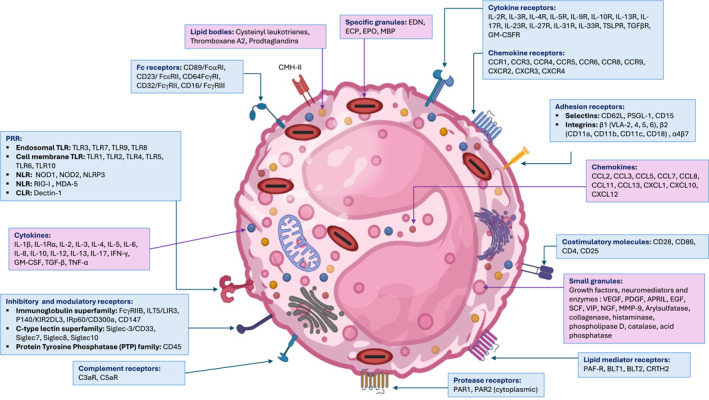
Schematic representation of eosinophil ultrastructure. Eosinophils are complex cells with a variety of membrane molecules and cellular mediators that enable them to function effectively in their microenvironment. They express several receptors for PAMPs/DAMPs, Immunoglobulins, cytokines, chemokines, proteases, or lipid mediators, allowing them to carry out their migratory functions, sustain activity through survival signals, as well as interact with other immune cells. These interactions involve surface integrins and selectins. Upon activation, eosinophils release granule contents such as eosinophil‐derived neurotoxin (EDN), eosinophil cationic protein (ECP), major basic protein (MBP), eosinophil peroxidase (EPO), various cytokines and chemokines, and lipid mediators. These proteins are crucial in inflammatory conditions, including infectious, allergic, and thrombotic environments. Their activity is regulated by the expression of inhibitory receptors that limit their function (see text for details).

**FIGURE 3:**
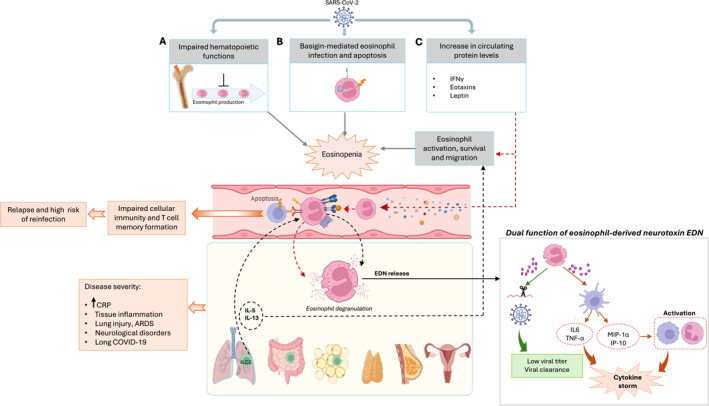
Eosinophil responses in COVID‐19 and possible factors leading to eosinopenia. Eosinopenia may result from SARS‐CoV‐2 effects on the hematopoietic functions of the bone marrow leading to reduced eosinophil production or eosinophil retention in the bone marrow (A). Apoptosis resulting from SARS‐CoV‐2 infection of eosinophils via basigin/CD147 may also contribute to the observed eosinopenia (B). Eosinopenia might also be due to the migration of blood eosinophils into tissues, driven by circulating mediators (such as IFN‐γ, eotaxins, and leptin) and cytokines secreted by tissue ILC2s (such as IL‐13 and IL‐5) (C). This process increases CD62L expression in eosinophils, enhancing their tissue infiltration and systemic inflammation. Once activated, eosinophils release their granule contents, particularly eosinophil‐derived neurotoxins (EDN), which can both contribute to the cytokine storm and tissue inflammation by recruiting innate immune cells and degrade viral RNA through its ribonuclease function. The high expression of programmed death‐ligand 1 (PD‐L1) on eosinophils in COVID‐19 could lead to lymphopenia by interacting with PD‐1 on T cells, potentially impairing memory cell production and increasing the risk of reinfection. Gray arrows denote possible factors resulting in eosinopenia, dashed arrows show the role of cytokines in eosinophil activation, survival and tissue migration, and solid black arrows demonstrate the distinct actions of EDN following its release. Orange arrows indicate the effects of eosinophil activation and tissue migration on disease severity.

Although crucial components of the body's immune system, eosinophils are implicated in the development of immunopathological conditions such as bronchial asthma, eosinophilic esophagitis, and hypereosinophilic syndromes.^74^ Recent studies revealed their additional functions in the defense against fungi, bacteria, and viruses by inducing or modulating immune responses through various mechanisms, including pattern recognition receptor (PRR) expression, release of granule content, or cytokine/chemokine secretion (Figure 2).^75^ It is noteworthy that a significant decrease in the number of eosinophils has been reported since the onset of the COVID‐19 pandemic. In the first studies conducted in Wuhan, over half of hospitalized COVID‐19 patients exhibited low eosinophil levels (<0.02 x 10^9 cells/L) upon admission.^27^ Similarly, a retrospective review of medical records for 85 COVID‐19 fatalities revealed that 81% of the patients had eosinophil counts below the normal range at the time of admission.^29^ The decline in eosinophil counts is particularly more pronounced in COVID‐19 patients when compared to both control subjects and those infected with other viruses.^9^, ^76^ The decrease in eosinophil levels was observed at least for infections involving the alpha and omicron variants,^77^, ^78^, ^79^ suggesting that it also occurred with intermediate variants and that it is a common feature of COVID‐19. Research on eosinopenia in COVID‐19 is limited, but it likely stems from various factors. These may include the suppression of eosinophil production, their retention in the bone marrow, or decreased functionality due to altered expression of certain molecules involved in their movement and activity.^5^, ^80^, ^81^ Other mechanisms may contribute to eosinopenia during COVID‐19, including the possibility that the virus can infect eosinophils. This infection may occur via basigin, also known as CD147, which has been identified as a co‐receptor for SARS‐CoV‐2.^82^, ^83^ Notably, basigin is highly expressed on circulating eosinophils in patients infected with SARS‐CoV‐2, whether moderately or severely ill. Interestingly, basigin expression is reduced on activated eosinophils from severely ill patients,^8^ which may reflect a reduction caused by viral uptake. While there is currently no direct evidence supporting this mechanism, these suggestions are supported by studies indicating that the virus utilizes basigin to infect platelets,^84^ leading to their apoptosis.^85^ Furthermore, the decrease in circulating eosinophils may also be associated with cell death induced by the release of proinflammatory cytokines including type 1, type 2 IFN and TNF‐α during acute infection, which may influence eosinophil apoptosis and modulate their activity.^71^, ^86^, ^87^, ^88^ Finally, eosinopenia may result in alteration of the production or the retention of eosinophils in the bone marrow as it was shown that certain SARS‐CoV‐2 variants, including alpha (B.1.1.7), delta (B.1.617.2), and omicron (BA.1.18/B.1.1.529.1.18), disrupt the hematopoietic functions of the bone marrow.^89^ These hypotheses are discussed below (Figure 3).

During the acute phase of the disease, eosinophils are one of the key cell populations responding actively, reflecting their early participation in antiviral defense. An activated phenotype in eosinophils has been observed in COVID‐19 patients upon admission, characterized by increased expression of CD69 and CD62L.^8^ Increased expression of IFN‐γ stimulates the expansion of resident rather than circulating eosinophils expressing CD62L during the first 6 days following infection.^71^ Subsequently, acute lung injury occurs 1 week later in severe patients.^71^ These observations are further supported by a strong correlation between eosinophil activation and both the SOFA score and maximum oxygen requirements.^8^ Hence, IFN‐γ might play a role in recruiting eosinophils to lung tissue, potentially triggering the hyperinflammatory state and ARDS observed in severe COVID‐19 patients around one week after admission. Given that IFN‐γ increases CD62L expression in eosinophils,^90^ examining the levels of IFN‐γ/CD62L expression could potentially predict the severity of lung injury in COVID‐19 patients. Similarly, a mouse model of respiratory syncytial virus (RSV) infection revealed a correlation between eosinophil infiltration in the airways and IFN‐γ expression.^91^ Additionally, elevated IFN‐γ levels have been observed in the nasopharyngeal secretions of infants with RSV infection.^92^ Moreover, the development of pulmonary lesions in COVID‐19 patients might result from the recruitment of CD62L^low^ inflammatory eosinophils, as observed in a RSV mouse model where allergic induction led to the recruitment of such eosinophils in an IL‐5‐dependent manner.^93^ Specifically, the lungs of IL‐5 transgenic mice, which had higher eosinophil levels, exhibited faster clearance of RSV owing to eosinophils' antiviral activity.^94^

Examination of lung biopsies and bronchoalveolar fluids in COVID‐19 patients revealed increased proinflammatory macrophage activity, characterized by a Th1 and Th17 inflammatory profile, while eosinophils were notably absent.^95^, ^96^ Reports suggest that a type 2 immune response, characterized by type 2 cytokines and eosinophil accumulation, might offer strong protection against COVID‐19.^97^ This achievement is attributable to eosinophil granule proteins, including eosinophil cationic protein (ECP), which is increased in the lungs of severe COVID‐19 patients.^98^, ^99^ Interestingly, ECP is also increased in nasopharyngeal secretions of infants infected with RSV or parainfluenza virus, highlighting its role as an antiviral mediator. These findings suggest the involvement of Th2 cells in eosinophil recruitment and activation, leading to local production of IL‐4 and IL‐5.^100^, ^101^ Eosinophil‐derived neurotoxins (EDN), which belong to the RNase A superfamily in humans, are also stored in eosinophil‐specific granules and released upon activation. These ribonucleases have the capability to degrade single‐stranded RNAs (ssRNAs) and may play a role in antiviral defense against SARS‐CoV‐2.^102^ Previous studies have shown increased ribonuclease activity in the bronchial secretions of patients infected with RSV.^103^ Likewise, recombinant human EDN demonstrated a dose‐dependent reduction in RSV infectivity on cultured respiratory epithelial cells in vitro.^104^ While exhibiting antiviral properties, EDN may also contribute to the inflammatory response seen in severe COVID‐19 cases. Indeed, EDN can stimulate dendritic cells to produce proinflammatory cytokines such as IL‐6 and TNF‐α, along with chemokines like MIP‐1α (CCL3) and IP‐10 (CXCL10), which attract macrophages and neutrophils. Additionally, it can induce the recruitment of eosinophils via chemokines like CCL5, which have been associated with disease severity in SARS‐CoV‐2 infection.^2^, ^105^, ^106^, ^107^ These findings are supported by the fact that high levels of EDN have been detected in the respiratory tract of patients with severe SARS‐CoV‐2 infection^99^, ^101^ but also by the correlation between the activated eosinophil phenotype in COVID‐19 patients and higher levels of soluble inflammatory markers, including CCL2, CCL7, CXCL10, IL‐18R1, and IFN‐γ.^8^

Eotaxin has been identified as one of the key cytokines distinguishing severe cases of COVID‐19.^108^ Eotaxin is a member of a chemokine family which includes CCL11/Eotaxin‐1, CCL24/Eotaxin‐2, and CCL26/Eotaxin‐3 and interacts with various receptors expressed on immune cells, notably CCR2, CCR5, and CCR3. These receptors, especially CCR3, are highly expressed in eosinophils, facilitating their trafficking and chemotaxis.^109^, ^110^ Hence, SARS‐CoV‐2 infection may induce eosinophil migration in the lung, thus explaining the low circulating eosinophil count. IL‐5 and IL‐13, continually produced and sustained by resident type 2 innate lymphoid cells (ILC2) are also crucial for eosinophil survival and recruitment.^111^ Interestingly, these cytokines are overexpressed in severe COVID‐19 patients^108^ and may contribute, together with eotaxin, to the accumulation of eosinophils in the lungs.^112^ Taken together, these data clearly indicate that during SARS‐CoV‐2 infection, eosinopenia could be the result of intense eosinophil migration and accumulation in the lung rather than eosinophil depletion. Eosinophils may then mediate persistent inflammation. Indeed, elevated levels of CCL11/eotaxin‐1 have been reported beyond 28 days post‐infection^113^ and may be one of the causes of developing long COVID and the onset of neurological disorders, as demonstrated in a mouse model.^114^ Likewise, eosinophils can contribute to cardiac and pulmonary complications leading to severe illness or death, such as acute eosinophilic pneumonia, eosinophilic granulomatosis, eosinophilic vasculitis,^115^ polyangiitis, and eosinophilic myocarditis.^116^ However, some consider that eosinophilia during COVID‐19 is associated with reduced inflammation described by low C‐reactive protein levels, probably reflecting a protective role of eosinophil persistence in the blood.^117^ Therefore, given that eosinopenia is most commonly associated with a poor prognosis,^118^, ^119^ the decrease in circulating eosinophil levels suggests migration to tissues and the initiation of tissue inflammation in response to cytokine signals^120^ or indirectly under the action of hormones such as leptin,^121^ which characterize patients at high risk of developing a severe form of COVID‐19.^122^, ^123^

Precise immunophenotypic patterns related to diagnosis and severity have been identified through immunophenotyping of blood leukocytes using multiparametric flow cytometry.^5^, ^8^, ^124^ Compared with negative controls, eosinophils from COVID‐19 patients exhibited downregulation of the prostaglandin D2 receptor, CRTH2. This may result in a less activated eosinophil profile and reduced pathogen clearance during viral infection. SARS‐CoV‐2 infection may thus be associated with inhibition of Th2‐polarized immune responses and decreased chemotaxis of CRTH2^+^ cells, thereby promoting the pathophysiology of post‐COVID‐19 pulmonary fibrosis.^5^ In addition, in patients with severe COVID‐19, CD69^+^ eosinophils exhibited a limited functional profile with reduced expression of CD66b, CD147, and CD11a, indicating a post‐degranulation state.^8^ Hence, immunophenotyping could be used to describe a predictive granulocytic signature of patients at high risk of developing a severe form of COVID‐19.^5^ Beyond the disturbances seen during the acute phase, eosinophil dynamics are also affected in the recovery phase, indicating extended immune disruption.^124^ This underscores a potential role in shaping the course of the disease, influencing symptomatology and overall prognosis.

Programmed death‐ligand 1 (PD‐L1) expression was significantly higher in eosinophils in patients with severe COVID‐19^5^ and a positive correlation between CD69^+^ eosinophils and soluble PD‐L1 expression has been observed.^8^ PD‐L1 expression by eosinophils is dependent on IFN‐γ^87^ and patients with severe COVID‐19 have elevated levels of IFN‐γ.^108^ Strikingly, during SARS‐CoV‐2 infection, eosinophil levels are correlated with lymphocyte counts.^27^ This suggests that an interaction is possible between these two cell types involving an effect on each other. Considering the studies reporting that CD4 T cells overexpress PD‐1 in severe COVID‐19 patients and those also evoking a high expression of PD‐L1 on eosinophils,^2^, ^5^, ^125^ it is tempting to speculate that the interaction of PD‐1 on T cells with its ligand expressed on eosinophils will inhibit the activation of T lymphocytes and promote their exhaustion and apoptosis, explaining the lymphopenia seen in COVID‐19 patients and its persistence in severe forms.^6^

Overall, the exact function of eosinophils during SARS‐CoV‐2 infection and COVID‐19 remains unclear, but it is crucial to note that they do not consistently contribute to respiratory and systemic illnesses. Therefore, it is imperative to investigate the role of these granulocytes to prevent potential pathophysiological effects or enhance immune responses that may help combat complications associated with SARS‐CoV‐2.

## 4 COVID‐19 AND ASTHMA: THE EOSINOPHIL CONNECTION

Asthma, a chronic inflammatory condition of the airways, is marked by heightened reactivity, mucus secretion, and reversible airway obstruction.^126^ In asthma patients, eosinophils comprise at least 60% of the granulocytes found in the airways.^127^ Respiratory viruses like RSV can worsen asthma. Interestingly, eosinophils in the respiratory tract may have antiviral effects, but during allergies, their activation and abundance could cause tissue damage, worsening the immune response.^128^ This underscores the importance of studying COVID‐19 in the asthmatic population. Remarkably, few to no cases of asthma or allergic conditions such as atopic dermatitis or rhinitis have been reported in a cohort of patients with mild or severe COVID‐19 illness.^27^, ^129^ Others considered that having allergic conditions was linked to a higher likelihood of being diagnosed with COVID‐19 but lower mortality rates post‐infection.^130^ Interestingly, an Italian study examining medical records from 96,149 deceased patients did not find any allergic conditions listed among associated diseases despite their prevalence in the general population.^131^ This might indicate a protective role of asthma against the virus or against the development of severe or symptomatic forms associated with SARS‐CoV‐2 infection. Asthma results from persistent inflammation of the bronchial tubes, primarily driven by type‐2 inflammation involving eosinophils, basophils, mast cells, Th2 helper cells, IgE‐producing B cells, and ILC2. This immune response is stimulated by cytokines like IL‐4, IL‐5 and IL‐13, leading to elevated eosinophil counts and antibody levels.^132^ Allergic asthmatic individuals may experience a less severe form of COVID‐19 as certain cytokines (IL‐4 and IL‐13) diminish ACE2 activity in the respiratory tract.^133^ In addition, individuals with asthma are typically accompanied by higher peripheral blood eosinophil counts and might experience a more favorable prognosis.^134^ Numerous studies have affirmed that individuals with asthma under good control do not face elevated susceptibility to being infected with COVID‐19.^135^ However, asthmatic individuals are at a high risk of developing respiratory signs associated with COVID‐19 and long COVID disease.^135^, ^136^ Moreover, the occurrence of SARS‐CoV‐2 infection in asthmatic patients may compromise asthma control after recovery.^137^

It has been found that the immune response to SARS‐CoV‐2 infection primarily involves Th1 and Th17 cell types.^138^, ^139^ Specific Th2 response did not show consistent findings, and gave rise to conflicting results since several studies indicated the lack of a type 2 immune response and consequent eosinophil activation, while others linked it to disease severity.^108^, ^138^, ^140^ The reduced eosinophil count in COVID‐19 patients contrasts with their abundance in allergic individuals with asthma and rhinitis, suggesting a potential protective role of eosinophils in the immune response against the virus, mitigating severe outcomes.^141^ Activated eosinophils in asthma may exhibit antiviral activity through degranulation, releasing ribonucleases like EDN, which directly target viral ssRNA, constituting a potential mechanism for viral suppression.^141^

It appears that type‐2 cytokines can in turn mitigate immunopathology in asthmatic individuals. IL‐13 is mainly secreted by activated Th2 cells but also by eosinophils and basophils. It regulates inflammatory and immune responses by suppressing macrophage activity, thereby reducing the production of pro‐inflammatory cytokines and chemokines.^142^ We can hypothesize that IL‐13‐secreting eosinophils might influence the cytokine storm and decrease the hyperinflammatory state associated with the severity and mortality of SARS‐CoV‐2 infection. Consequently, this effect may influence the polarization of alveolar macrophages toward an M2‐like profile, potentially mitigating inflammation and lung damage.

Several other mechanisms could also explain the low susceptibility of the asthmatic population to the severe form of COVID‐19. Indeed, the SARS‐CoV‐2 shares genetic sequences with certain allergens.^133^, ^143^ By retaining the memory of the initial encounter, their inflammatory response during an infection could be more moderate or less pronounced.^144^ Furthermore, it has been shown that sensitizing mice to an allergen provides an advantage when challenged with parainfluenza virus by reducing viral RNA due to the increased presence of eosinophils in the lungs.^127^ Moreover, the reduced likelihood of developing severe forms of COVID‐19 in asthmatic individuals can be attributed to the use of corticosteroids as a foundational treatment.^145^, ^146^ In addition, biological antagonists targeting the type‐2 response (omalizumab, benralizumab, dupilumab, mepolizumab, reslizumab) could potentially modulate the dysregulated immune response and provide protection against severe COVID‐19 complications in asthmatic infected individuals.^12^ Unlike healthy subjects, eosinophils in asthmatic patients do not exhibit the ability to capture viruses,^147^ which might provide an advantage in the context of COVID‐19 by restricting eosinophil activation and preventing eosinophil‐dependent viral dissemination to the tissues. Nevertheless, environmental elements such as tobacco use, the type and severity of asthma, adherence to medication, and concurrent health conditions could impact the overall risk.^148^

## 5 SARS‐CoV AND SARS‐CoV2 VACCINES AND PULMONARY EOSINOPHILIA

After the emergence of SARS‐CoV, various approaches were explored to create potential vaccines. Those candidates which elicited neutralizing antibodies against the S protein showed effectiveness in inhibiting viral replication.^149^ Although SARS‐CoV vaccines (such as live attenuated and inactivated virus vaccines, which have not been tested in humans) utilize a different technology than COVID‐19 vaccines (predominantly mRNA‐type), post‐vaccination observations provide valuable insights regarding eosinophil responses.^150^, ^151^ In multiple animal models, vaccination against SARS‐CoV has been linked to pulmonary eosinophilia, showcasing an intensified immune reaction characterized by eosinophil infiltration in the lungs following exposure to virus replicon expressing structural proteins (S or N) or an inactivated virus vaccine in previously immunized animals.^151^, ^152^, ^153^, ^154^ In betacoronaviruses, specific sequences of the nucleocapsid protein (N) appear to be responsible for the development of eosinophilic pathologies, underscoring the importance of taking them into account in vaccine design strategies.^151^

**FIGURE 4:**
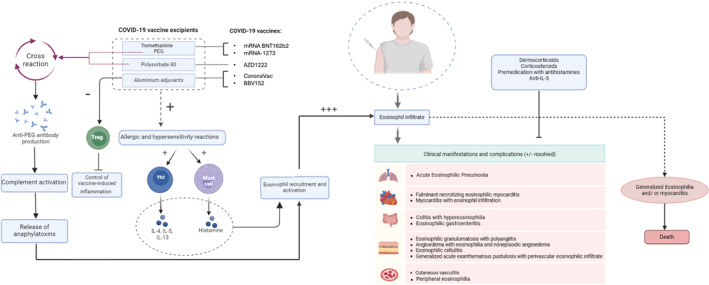
Involvement of eosinophils in the post‐COVID‐19 vaccination phase. COVID‐19 vaccines can cause clinical manifestations following the first dose or booster doses. Eosinophilic infiltrates are associated with cutaneous complication or organ involvement, which can lead to generalized eosinophilia and potential death. Whole inactivated virus vaccines that use aluminum as an adjuvant are particularly prone to causing these clinical issues due to the ability of these adjuvants to stimulate Th2 immunity, promoting the survival and tissue infiltration of eosinophils while inhibiting the action of Tregs in controlling inflammation. The mRNA vaccines include excipients, such as tromethamine and polyethylene glycol (PEG), that can trigger allergic and hypersensitivity reactions.^167^, ^185^ Polysorbate 80 in the AstraZeneca vaccine may cross‐react with PEG,^181^ leading to the production of anti‐PEG antibodies.^182^ This, in turn, activates the complement system, releasing anaphylatoxins. These molecules promote eosinophil recruitment, activation, and tissue infiltration, further intensified by the activation of other cells involved in allergic and hypersensitivity responses, such as Th2 cells and mast cells. Dermocorticoids, corticosteroids, anti‐IL‐5 and antihistamines have been used to solve such complications.

Regarding SARS‐CoV‐2, the Pfizer‐BioNTech vaccine was the first vaccine to receive temporary regulatory approval on 2 December 2020^155^ and other vaccines were subsequently developed.^156^ Eosinophilic manifestations have also been observed following the administration of anti‐SARS‐CoV‐2 vaccines,^157^, ^158^, ^159^ summarized in Table 1 and Figure 4. Eosinophilic pneumonia has been reported in a 55‐year‐old woman with no particular history of comorbidity or associated infectious diseases, 7 weeks after receiving the first dose of the ChAdOx1 nCov‐19 COVID‐19 vaccine.^161^ Similarly, vaccination with Sinovac/CoronaVac COVID‐19 vaccine resulted in a pulmonary complication manifested by pulmonary eosinophilia in a 73‐year‐old non‐smoking patient, probably linked to the aluminum‐based adjuvants.^163^ Interestingly, vaccination with aluminum adjuvants triggers a Th2‐mediated immune response, which actively contributes to the development and maturation of eosinophils through the release of various cytokines such as IL‐3, IL‐5 and GM‐CSF. In turn, aluminum adjuvant‐based vaccines do not stimulate Treg cells to regulate robust Th2‐mediated immune responses.^183^ Four days after receiving the mRNA vaccine (Pfizer‐BioNTech), a 64‐year‐old man developed generalized acute exanthematous pustulosis characterized by a perivascular eosinophilic infiltrate.^157^ Another case of eczema with eosinophilic infiltrate has also been reported in an 80‐year‐old individual, occurring 8 days after receiving the first dose of Pfizer vaccine and 4 days after the second dose. Despite initial treatment with an anti‐histamine or anti‐fungal agent that did not yield favorable results, administration of a dermocorticoid was necessary.^177^ Other cutaneous manifestations have occurred following vaccination with viral vector vaccines (AstraZeneca), such as erythema nodosum in 3 individuals aged over 45, who were subsequently treated with dermocorticoids and anti‐histamines.^178^ Various cutaneous manifestations associated with eosinophilic infiltration have also been reported in patients vaccinated with different types of COVID‐19 vaccines (not limited to mRNA vaccines), including eosinophilic granulomatosis with polyangiitis,^158^, ^165^, ^178^ angioedema with eosinophilia,^164^, ^174^ and cutaneous vasculitis.^115^ Beyond cutaneous involvement, vaccination has also been linked to eosinophilic involvement in pulmonary and gastrointestinal tissues.^161^, ^173^, ^176^, ^177^, ^183^ Abundant eosinophilic infiltrates have been observed in autopsies following COVID‐19 vaccination, most often associated with cardiovascular complications,^160^, ^162^, ^171^, ^172^ but also localized in different cellular compartments of the body, that is, pulmonary, intestinal, renal and cerebral.^184^ Another instance of generalized eosinophilia leading to death has also been reported following vaccination.^168^ Regardless of age or gender, the anti‐SARS‐CoV‐2 vaccine appears to potentially trigger an eosinophilic immunopathological response, as children can also be affected (eosinophilic cellulitis and myocarditis).^172^, ^175^ In the context of SARS‐CoV, Bolles et al. indicated that this immunopathological impact correlates with the inclusion of the N protein in the vaccine.^151^ The eosinophil‐driven pathogenesis induced by SARS‐CoV‐2 may be associated with increased activation, leading to the secretion of IL‐4, IL‐13, and eotaxin, which in turn stimulate Th2‐type responses.^152^ These responses appear to be significant risk factors for severity and adverse outcomes.^185^ In a mouse model of immunization against SARS‐CoV, it was shown that incorporating a Toll‐like receptor agonist into vaccine formulations leads to the production of high levels of neutralizing antibodies against SARS‐CoV while also preventing pathogenic eosinophil infiltration in the lungs.^152^ Eosinophilic infiltration‐related damage following COVID‐19 vaccination may also be linked to the presence of excipients, which can trigger allergic reactions, including severe cases such as anaphylactic shock.^167^, ^182^, ^189^ Indeed, a direct cause of hypersensitivity reactions following Moderna COVID‐19 vaccination has been attributed to tromethamine, an excipient present in this mRNA vaccine.^180^ Similarly, polyethylene glycol (PEG), found in the lipid nanoparticles encapsulating mRNA in vaccines like Pfizer and Moderna, has been implicated in such reactions.^167^, ^190^ Beyond eosinophil activation, PEG may also compromise vaccine efficacy by inducing the formation of antibodies.^188^ In this regard, PEG can stimulate the release of pre‐existing or newly formed anti‐PEG antibodies, leading to complement activation.^181^, ^186^ This process generates the anaphylatoxins C3a and C5a, which recruit and activate eosinophils, as well as other cells such as mast cells and endothelial cells. The actions of these anaphylatoxins create a favorable environment that facilitates eosinophil infiltration.^181^, ^191^ Similarly, the AZD1222 (Oxford‐AstraZeneca) vaccine contains polysorbate 80, which can cross‐react with PEG.^181^ Although these reactions are rare, both PEG and polysorbate 80 remain potential risk factors for individuals with a history of immediate allergic reactions (immediate hypersensitivity) or for those with undiagnosed allergies.^191^ Indeed, cases of peripheral eosinophilia have been reported in patients with a history of allergies or asthma following mRNA COVID‐19 vaccination (Moderna and Pfizer). This eosinophilia was associated with worsening respiratory symptoms, requiring treatment with anti‐IL‐5 agents and corticosteroids.^159^ In an ex vivo model of eosinophilic asthma, the Pfizer COVID‐19 vaccine was found to enhance contractile sensitivity to histamine, potentially explaining the observed bronchospasm in asthmatic patients post‐vaccination, probably related to the presence of PEG.^192^ These findings indicate a significant activation of pulmonary eosinophils in response to elevated histamine levels as they utilize their histaminase content (Eosinophil diamine oxidase) for its breakdown.^194^, ^195^ Prolonged eosinophil activation, however, may induce tissue damage, potentially exacerbating clinical symptoms.^195^ This implies that ensuring the safety and efficacy of a candidate vaccine against SARS‐CoV‐2 requires considering the risk of immunopathogenicity associated with eosinophil infiltration, which may be reduced by modifying adjuvants,^152^, ^163^ using polysorbate 80‐, tromethamine‐ and PEG‐free vaccines and employing premedication with antihistamines or corticosteroids for patients sensitive to these components.^196^

**TABLE 1:** Main eosinophilic disorders associated with COVID‐19 vaccines.

Type of damage	Vaccine	Reference	Sex (n)	Age
Acute eosinophilic pneumonia	AZD1222 (Oxford‐AstraZeneca)^c^	160	Male (1)	66
	mRNA BNT162b2 (Pfizer‐BioNTech)^b^	161	Female (1)	55
	CoronaVac (Sinovac)^a^	162	Male (1)	37
		163	Female (1)	73
Eosinophilic granulomatosis with polyangiitis	mRNA‐1273 (Moderna)^b^	164	Male (1)	63
	mRNA BNT162b2 (Pfizer‐BioNTech)^b^	158	Female (1)	79
		165	Female (1)	71
Angioedema with eosinophilia	mRNA BNT162b2 (Pfizer‐BioNTech)^b^	166	Female (1)	26
Eosinophilic cellulitis	mRNA BNT162b2 (Pfizer‐BioNTech)^b^	167	Male (1)	12
Generalized eosinophilia	mRNA‐1273 (Moderna)^b^	168	Male^d^ (1)	76
Colitis with hypereosinophilia	mRNA BNT162b2 (Pfizer‐BioNTech)^b^	169	Male (1)	61
Cutaneous vasculitis	Inactivated viral vaccine BBV152 (COVAXIN)**^a^**	115	Female (1)	31
Fulminant necrotizing eosinophilic myocarditis	mRNA BNT162b2 (Pfizer‐BioNTech)^b^	170	Female^d^ (1)	57
Myocarditis with eosinophil infiltration	mRNA‐1273 (Moderna)^b^	160, 162, 171	Male^d^ (1,1,8)	42, 27,33
	mRNA BNT162b2 (Pfizer‐BioNTech)^b^	171	Female^d^ (6)	36
		174	Male^d^ (2)	Teens
		171	Male^d^ (8)	45
		173	Female (1)	45
Nonepisodic angioedema	AZD1222 (Oxford‐AstraZeneca)^c^	175	Female (1)	70
Eosinophilic gastroenteritis	mRNA‐1273 (Moderna)^b^	176	Male (1)	65
Generalized acute exanthematous pustulosis with perivascular eosinophilic infiltrate	mRNA BNT162b2 (Pfizer‐BioNTech)^b^	157	Male (1)	64
Eczema with eosinophilic infiltrate	mRNA BNT162b2 (Pfizer‐BioNTech)^b^	177	Male (1)	80
Erythema nodosum	AZD1222 (Oxford‐AstraZeneca)^c^	178	Female (3)	66, 62, 46

^a^ Denotes vaccines with aluminum adjuvants.

^b^ Denotes vaccines with PEG and tromethamine.

^c^ Denotes vaccines with polysorbate 80.

^d^ Denotes a deceased individual.

## 6 CONCLUSION

SARS‐CoV‐2 affects all organ systems and immune compartments, leading to a notable decline in eosinophil counts, which seems to be multifactorial. Eosinophils play a crucial role in the immune response during the acute phase of COVID‐19, displaying functional changes that categorize them as cells with dual or contradictory functions. This duality arises from their ability to mediate inflammation through degranulation or secretion of pro‐inflammatory mediators while influencing other immune cells. Additionally, eosinophils may compromise adaptive responses through intercellular interactions with T lymphocytes via the PD‐1/PD‐L1 complex.

Moreover, degranulation can have antiviral properties, helping to prevent viral replication through the action of RNAses such as EDN and ECP. Post‐vaccination eosinophilic disturbances have been linked to various organ complications, which have been managed with corticosteroids, antihistamines, and anti‐IL‐5 therapies. This suggests that eosinophils significantly contribute to inflammation during both vaccination and potentially during natural infection.

## CONFLICT OF INTEREST STATEMENT

JV reports speaker and consultancy fees in the past 5 years from Astra Zeneca, HpVac, L’Oréal, Novartis, Sanofi, Thermo Fisher Scientific, and travel expenses reimbursement from Stallergènes‐Greer for an international meeting outside the submitted work. The other authors declare no conflicts of interest.

## AUTHOR CONTRIBUTION

WS: Performed search and wrote the manuscript; JV, BD: Designed project, wrote the manuscript, reviewed the manuscript; BD: supervised process.

## Data Availability

Data sharing is not applicable to this article as no new data were created or analyzed in this study.
