# Correction to “Eosinophils and COVID‐19: Insights into immune complexity and vaccine safety”

**DOI:** 10.1002/clt2.70056

**Published:** 2025-04-14

Sahli W, Vitte J, Desnues B. Eosinophils and COVID‐19: insights into immune complexity and vaccine safety. *Clin Transl Allergy*. 2025;e70050.

In Figure 1, the icons of monocyte and T cell and some labels were missing. This should have appeared as the following Figure 1.


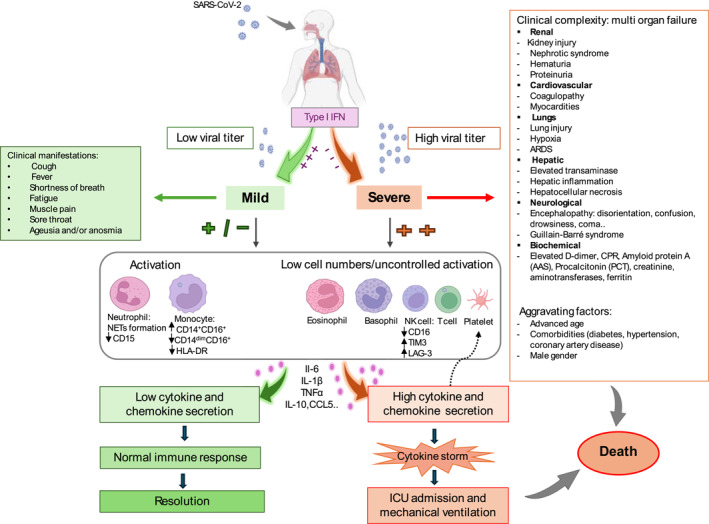


We apologize for this error.

